# Dexmedetomidine and Fentanyl Exhibit Temperature Dependent Effects on Human Respiratory Cilia

**DOI:** 10.3389/fped.2015.00007

**Published:** 2015-02-11

**Authors:** Nils Welchering, Sebastian Ochoa, Xin Tian, Richard Francis, Maliha Zahid, Ricardo Muñoz, Cecilia W. Lo

**Affiliations:** ^1^Department of Pediatrics, University of Pittsburgh, Pittsburgh, PA, USA; ^2^Office of Biostatistics Research, NHLBI, Washington, DC, USA; ^3^Department of Developmental Biology, University of Pittsburgh, Pittsburgh, PA, USA; ^4^Department of Critical Care Medicine, University of Pittsburgh, Pittsburgh, PA, USA

**Keywords:** dexmedetomidine, ciliary beat frequency, hypothermia, human cilia, fentanyl, airway cilia motility

## Abstract

**Background:** Dexmedetomidine (dex) is commonly used in intensive care due to its effective sedation and analgesia with few adverse effects and minimal respiratory depression. However, we recently observed that exposing mouse epithelial respiratory cells to dex decreased ciliary beat frequency (CBF), suggesting dex may pose pulmonary risk.

**Objective:** The purpose of this study is to determine the effects of dex at clinically relevant doses on CBF in human respiratory epithelia.

**Methods:** Human nasal epithelial cilia were obtained from the inferior nasal turbinate with a rhinoprobe and placed in culture medium at 15°C and 37°C. At 5 and 30 min, video-microscopy was used to assess CBF, either without (control) or with different concentrations (1, 5, and 10 nM) of dex, fentanyl (fen), and dex + fen combination.

**Results:** At 15°C, CBF was lower in the dex group compared to controls at 5 and 30 min. At 37°C, there was a significant increase in CBF with dex at 5 and 30 min, except for dex at 5 nM after 5 min, which showed a significant decrease. At 15°C the combination of dex + fen showed a positive interaction, causing less ciliary inhibition as expected. In contrast, no interaction between drugs was seen between dex and fen at 37°C.

**Conclusion:** At low temperatures, dex reduces CBF in human respiratory epithelia, whereas dex increases CBF at physiologic temperature *in vitro*. Whether these effects translate into clinical consequences during hypothermia, as with cardiopulmonary bypass surgery will require further studies.

## Introduction

In intensive care medicine, physicians face the challenge of providing effective sedation while minimizing adverse effects. Sedatives are necessary to provide patient comfort and safety, but their administration must be weighed against deleterious consequences. Dexmedetomidine (dex), an alpha-2 agonist, is being increasingly used due to its effective sedative property (1). This has made dex appealing in cardiac intensive care, where hemodynamic instability warrants medications that minimally compromise cardiovascular and respiratory function, especially during postsurgical care after cardiopulmonary bypass surgery (CBP) (2, 3). However, we recently obtained data from *in vitro* studies of the mouse respiratory epithelia showing dex decreased ciliary beat frequency (CBF), an objective measure of ciliary motility, thereby raising concerns about dex compromising lung clearance function. CBF plays an essential role in propelling mucus and foreign particles out of the airways. Although studies have identified a multitude of factors that can depress CBF, including anesthetics and hypothermia, the effects of dex on human respiratory epithelia have not been specifically examined (4–6). Therefore, we undertook the present study to determine the effects of dex on ciliary motility at 15°C and 37°C in humans.

## Materials and Methods

### Obtaining human respiratory epithelia

This study was approved by the University of Pittsburgh Internal Review Board. Informed consent was obtained from all volunteers. Human nasal epithelial cells from 10 adult volunteers were collected with a rhinoprobe curette (Arlington Scientific, Springville, UT, USA) using a nasal speculum. The curette was gently scraped three times over the surface of each inferior nasal turbinate and the tissue was immediately placed in 5 ml RPMI-1640 culture medium (Leibowitz-15 medium) at room temperature. Tissue obtained from one person was used as a single sample.

### Processing of human respiratory epithelia and preparation of cell groups

Samples from the same individual were placed in media precooled or heated at 15 or 37°C, immediately after extracting the samples from healthy volunteers. Video-microscopy was performed 5 and 30 min after placing the cells in the media, and CBF calculated from these videos. Media contained either no drug (control group), or an anesthetic drug at 1, 5, and 10 nM concentration comprising: (a) dex, (b) fentanyl (fen), or (c) dex + fen combination. These concentrations are equivalent to therapeutic plasma concentrations achieved during the course of routine clinical care (7, 8). To ensure similar sampling from each subject for each condition, tissue from five different volunteers was obtained and tested across all conditions for a total of 1991 data points.

### Measurement of ciliary beat frequency

Ciliary motion was recorded with a Leica DMIRE2 inverted microscope (Leica Microsystems, Buffalo Grove, IL, USA) using a 100× differential interference contrast (DIC) oil objective under DIC optics. To achieve a constant temperature of 15°C, the microscope was placed in a room with an adjusted temperature of 15°C. For 37°C, the microscopy was conducted with a heated objective maintained at 37°C (Bioptechs, Butler, PA, USA). Videos were captured at 200 frames/s with a Phantom v4.2 high speed CMOS camera (Vision Research, Wayne, NJ, USA). To measure CBF, at least four videos were recorded for each condition. The videos obtained were analyzed with ImageJ (NIH) to create a kymograph for measuring the CBF. The CBF was measured from eight regions of interest showing beating cilia per video and averaging five successive wavelengths per region using GIMP v2.6. Mean CBF was calculated for the control group as well as for dex, fen, dex + fen at both temperatures and time points.

### Real time PCR analysis

Nasal tissue was placed in culture on rat tail collagen-coated plates and media containing Ultroser G (Pall Corp.), and grown to confluence. Once confluence was achieved, collagen was removed using collagenase IV (Worthington Biochemicals, NJ, USA) and cells placed in suspension on a 37°C orbital shaker. The nasal epithelial cells lose their cilia as they grow on the collagen substratum, and when placed in suspension culture, they reciliate within 10–14 days (9). Once reciliation was confirmed, RNA from these cells was extracted using RNeasy Plus micro kit (Qiagen, cat #74034), with generation of amplified cDNA (NuGen, Ovation RNA-Seq System, cat #7102-32), and real time PCR (rt-PCR) performed using primers specific for human alpha and beta-adrenergic receptors as well as opioid receptors. We also isolated nasal epithelial cells using a rhinoprobe to scrape the inferior turbinate under direct visualization using a nasal speculum. Cells were lysed using RNA lysis buffer (Qiagen), reverse transcription performed to generate cDNA and rt-PCR performed on this sample using the same primers as above for human alpha and beta-adrenergic as well as opiod receptors. All expression levels were normalized against human beta-actin gene.

### Statistical analysis

Differences in the mean CBF values between each drug category and control and difference between two temperatures for each drug or control were determined by heteroscedastic unpaired *t*-tests. A multivariate linear regression model was constructed to assess the main effect of the two drugs (dex and fen) and their interaction effect, separately, for each temperature. At each temperature, a regression model was fitted to the CBF data from the three drugs with control as the reference group. The intercept in the model is the estimated mean CBF for the control; the other positive or negative regression coefficients represent estimated increases or reductions in CBF for individual drugs and their interaction as compared to the control. The mean effect of the drug concentrations (1, 5, or 10 nM) and time of exposure to drug (5 or 30 min) were also examined using the regression model. All tests were two-tailed and *p*-values <0.05 were considered significant. Analyses were performed with SAS 9.3 (SAS Institute, Cary, NC, USA).

## Results

In order to evaluate the effects of dex and fen on respiratory airway cilia, human nasal epithelial cells were obtained by curettage of the inferior nasal turbinate under direct visualization video-microscopy performed. Cells from 10 volunteers (6 male, 4 female, median age 30) were obtained and a total of 1991 videos were obtained for this study (Table S1 in Supplementary Material).

### Effects of temperature on ciliary beat frequency

**Figure 1:**
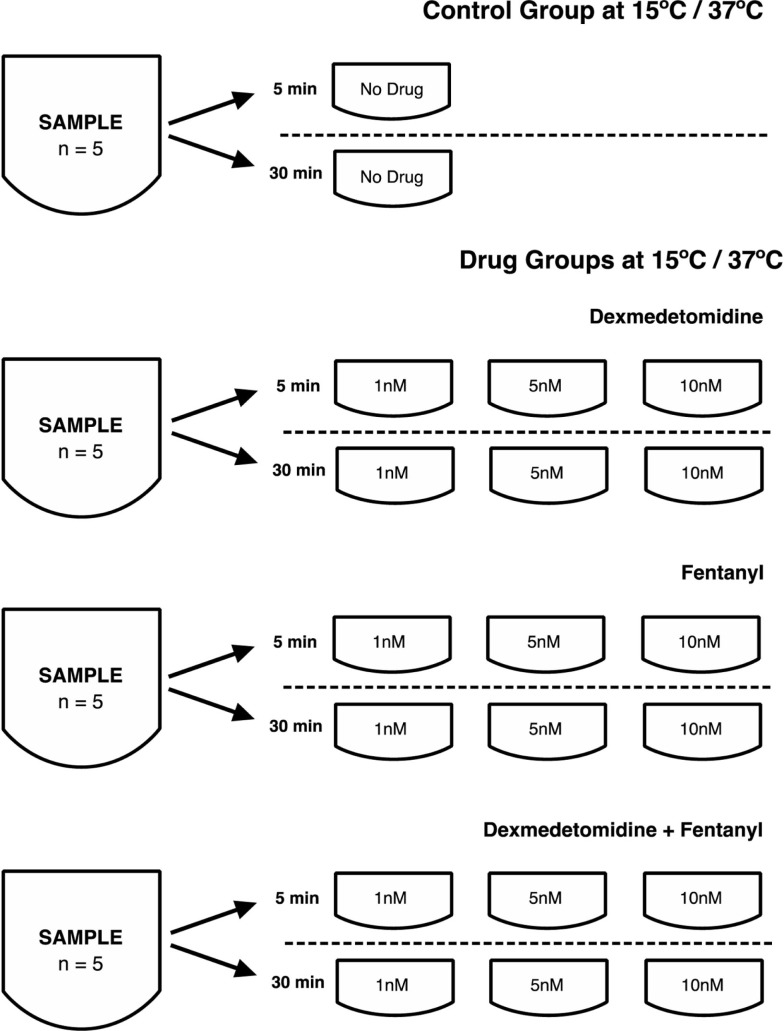
**Processing of human respiratory epithelia and preparation of cell groups**.

We examined temperature effects on CBF at 15°C to mimic conditions during CPB surgery and 37°C corresponding to body temperature. The CBF was obtained with exposure to temperature and drugs at 5 and 30 min, with samples exposed to drugs including dex, fen, and dex + fen at 1, 5, and 10 nM drug concentrations (Figure 1). Mean CBF was significantly lower in all groups at 15 vs. 37°C at 5 and 30 min (Table S2 in Supplementary Material).

### Effects of drug exposure on ciliary beat frequency

**Figure 2:**
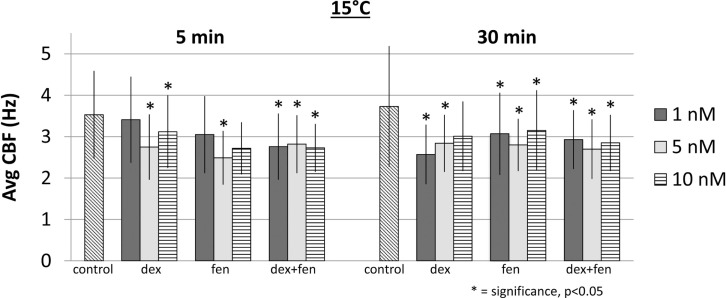
**Effects of drug exposure on ciliary beat frequency at 15°C**.

**Figure 3:**
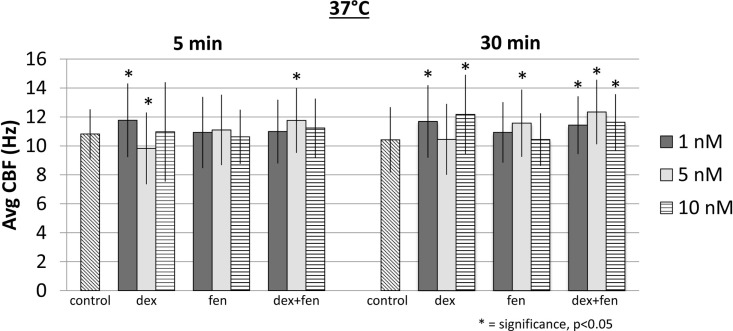
**Effects of drug exposure on ciliary beat frequency at 37°C**.

At 15°C, exposure to drugs caused a reduction of CBF at all concentrations at 5 and 30 min. Figure 2 shows mean CBF for each group (*p* < 0.05). When compared to controls, all drugs led to a significant increase in CBF at 37°C, except for dex 5 nM at 5 min, which decreased CBF significantly (Figure 3). This unexpected cilia-depressant effect was no longer significant when the groups of dex with 5 nM concentration at 5 and 30 min at 37°C were pooled together and compared to controls.

### Drug–drug and drug–time interaction on ciliary beat frequency

To investigate drug interactions between dex and fen, a multivariate linear regression model was constructed separately for each temperature. Although there are differences in CBF values under different concentrations or drug exposure times for each given drug, the average effect of drug concentration across the three drugs was not significant in the regression models. The average effect of drug exposure time was significant in the model at 37°C, but not at 15°C. When using controls as reference, the combination of dex + fen showed a positive interaction at 15°C, causing less cilia inhibition than expected by summation of their individual effects. In contrast, no interaction was seen between dex and fen at 37°C. Thus the estimated effect for dex + fen group was similar to the sum of the individual positive effects from dex and fen (Table 1).

**Table 1:** **Drug–drug and Drug–time interaction on ciliary beat frequency**.

Variables	15°C (*n* = 818)		37°C (*n* = 1173)	
	Estimate (SE)	*p*-value	Estimate (SE)	*p*-value
Intercept	3.64 (0.11)	<0.0001	10.51 (0.30)	<0.0001
Fen	−0.76 (0.13)	<0.0001	0.28 (0.32)	0.37
Dex	−0.67 (0.13)	<0.0001	0.43 (0.33)	0.19
Dex and fen interaction	0.60 (0.15)	<0.0001	0.20 (0.37)	0.59
Drug exposure time (30 vs. 5 min)	–	–	0.31(0.14)	0.023

Multivariate linear regression model was also used to show time effects on the CBF, as the analysis of variance did not show effects between different concentrations in each group. Accordingly, we pooled the data from different concentrations together to generate larger sample sizes. The regression model showed no significance for the effect of time at 15°C (*p* = 0.663). In contrast, the time effect in the regression model was significant at 37°C (*p* < 0.05, Table 2). The individual means for each group showed a trend toward increased CBF at 30 min compared to 5 min for dex (*p* = 0.089) and dex + fen (*p* = 0.035).

**Table 2:** **Multivariate regression model at 37°C**.

Mean CBF in Hz ± SD	Control	Dex	Fen	Dex + fen
At 5 min	10.82 ± 1.71	10.82 ± 2.93	10.88 ± 2.25	11.35 ± 2.16
At 30 min	10.42 ± 2.26	11.39 ± 2.65	11.00 ± 2.13	11.81 ± 2.09
*p*-value	0.45	0.089	0.58	0.035

### Adrenergic and opioid receptor transcript expression in the mouse airway epithelia

To examine possible molecular mechanism for the drug–drug interactions as well as drug–temperature and time effects of dex and fen, we investigated expression of adrenergic and opioid receptor subtypes in reciliated human nasal epithelia with rt-PCR. Quantitative PCR showed high level of adrenergic beta-2 receptor expression followed by beta-1 receptors, without detectable expression of alpha-adrenergic or opioid receptors (Figure S1 in Supplementary Material). Quantitative PCR results carried out on freshly isolated nasal epithelial cells showed high levels of beta-adrenergic receptor expression (beta-1 > beta-2), without detectable alpha-adrenergic or opioid receptors, similar to our findings from the quantitative PCR carried out on the reciliating tissue.

## Discussion

In the intensive care setting, administration of anesthetic agents requires a thorough understanding of their pharmacodynamics and adverse effects. Dex, an alpha-2 agonist, has been extensively used for sedation and analgesia due to its favorable safety profile (10, 11). In a recent animal study, we found significant cilia-inhibitory effect with dex at 10 nM at 22–24 and 37°C, but no significant effect at 15°C (12). The cilia-inhibitory effect of dex was reversed by combining dex with fen, which by itself was cilia-stimulatory at all temperatures. Conversely, a Japanese study found no effect of dex at 10 and 100 nM on CBF using cultivated rat tracheal epithelial cells at 26.5°C (13). Given these conflicting results and lack of human studies, we investigated the effects of dex on cilia motility using human nasal epithelial cells.

Nasal epithelial cells are known to be satisfactory surrogates of tracheal and bronchial epithelial cells (14) and therefore bronchial muco-ciliary clearance (15). The CBF has proven to be strongly correlated with the efficacy of mucus transport, with a non-linear relationship noted such that even slight reductions in CBF can translate into significant impairment in muco-ciliary transport (15). To interrogate the effects of the various drugs on CBF, we used concentrations equivalent to therapeutic doses and examined all samples at physiological temperature and at 15°C, the latter to simulate hypothermia during CPB surgery. We added fen to replicate our mouse study. After exposing human respiratory epithelial cells to dex, fen, and dex + fen, we found a significant cilia-stimulatory effect at 37°C, but a significant cilia-inhibitory effect at 15°C with all drugs. This suggests that administration of dex at physiologic temperatures would not cause cilia inhibition. This finding is important because this drug is mainly administered in critically ill patients who frequently have abolished airway reflexes, and are predisposed to pulmonary morbidity. Our finding of a cilia-inhibitory effect at low temperatures would suggest these drugs might have unfavorable effects in hypothermic patients, such as those undergoing CPB surgery. If cilia motility can be restored with re-warming the patient, such risks may be minimal. Further studies are needed to examine the reversibility of the low temperature effects.

We did not find significant correlation between drug concentration and CBF (e.g., higher concentrations did not cause a greater magnitude change in CBF). Furthermore, no significant changes in CBF over time were observed at 15°C (e.g., no differences between 5 and 30 min). This suggests that dex and fen does not produce progressively more depressive effects over time with drug exposure in hypothermia. In contrast, there was a significant time effect with drug exposure at 37°C. Longer exposure with dex or dex + fen led to higher CBF, whereas fen alone showed no difference. This suggests dex enhances CBF over time with drug exposure at 37°C. However, as these agents are given over longer periods for sedation purposes both at 15 and 37°C, the long-term effect on CBF remains unknown and requires further studies. There were significant drug interactions between dex and fen at 15°C. This was indicated by our linear regression modeling, which showed non-additive effects, with less CBF inhibition observed then would be expected by summation of the individual drug effects. These findings suggest there may be overlap in the mechanisms of CBF regulation by drugs and temperature.

### Possible mechanism for effects of dex and fen on CBF

The cilia-inhibitory effects of dex and fen were significant at low temperatures, whereas we found a significant trend toward cilia-stimulation at 37°C (except for dex 5 nm at 5 min). Temperature augments CBF in a sigmoid fashion, from cilia stasis below 10°C to a near optimal rate at 32°C (16). Low temperatures have been shown to inhibit adenyl cyclase activity, reduce cAMP concentrations and alter PKC and CAMKII activity in ciliated cells (17, 18). Studies have shown alterations in adrenergic and opioid receptor affinity in response to different agonists during hypothermia (17). Hypothermia may favor receptor affinity and second messenger profile that potentiates the individual and combined effects of dex and fen, thus producing synergistic cilia inhibition between drugs and temperature. In contrast, higher temperatures may produce a receptor affinity/second messenger configuration that might have opposing effects on CBF (19).

While our rt-PCR analysis did not detect expression of alpha-adrenergic receptors in reciliating human airway epithelia, we have observed low-level expression of alpha-2 adrenergic receptor transcripts in RNA-seq analysis of whole human respiratory epithelia obtained from nasal scrapes (M. Zahid and C. Lo, unpublished observations). This would suggest dex could act through the alpha-2 adrenergic receptor to modulate CBF in the human respiratory epithelia. The classical alpha-2 pathway is known to cause Gi-mediated adenyl cyclase inhibition, which would cause reductions in cAMP levels and intracellular calcium (20). However, as the transcript abundance was low, the effects of dex may involve another pathway. It is reported that dex blocks muscarinic subtype 3 receptor in Xenopus oocytes and inhibits intracellular calcium elevation (21), but these results have not been described in human cells. Several studies have shown fen is antagonistic to the muscarinic 3 receptor (22–24). An alternative explanation is that these drugs could act through a different cell type not present in the reciliating respiratory epithelia used for our rt-PCR analysis. These cells may modulate CBF in the intact human respiratory epithelia via transmission of ions and/or second messengers by gap junction mediated cell–cell communication (25, 26). This also could account for the previous report that dex and fen did not alter CBF in cultured reciliated respiratory epithelial cells (13).

### Limitations

We note the ciliated respiratory epithelia used for our analysis were removed from autonomic neural signaling and vascular supply, both of which are known to regulate CBF *in vivo* and are affected by dex (16). To maximize potential clinical relevance, drug concentrations used in this study were equivalent to therapeutic doses and all samples were examined at physiologic temperature and conditions mimicking hypothermia during CPB. Further studies are needed to evaluate the effect of dex *in vivo*. As clinical outcomes were not examined in this study, our results should be considered hypothesis generating and further studies are needed to see how these *in vitro* observations of the effects of dex on CBF translate into being clinically relevant.

## Conclusion

Nasal epithelial cells are known to be satisfactory surrogates of tracheal and bronchial epithelial cells. In the present study, we found that dex and fen are cilia-inhibitory at 15°C, and have cilia-stimulatory effects on CBF at physiologic temperatures. Increasing dose and combining both agents with exposure up to 30 min did not produce additional changes in CBF. Combination of dex + fen showed positive interaction, causing less cilia inhibition at 15°C. In contrast, no interaction between dex and fen was observed at 37°C. Whether these effects translate into clinical consequences during hypothermia, as with CBP surgery, or if this can be reversed upon temperature normalization, will require further studies. In addition, further studies are needed to delineate the receptors and second messengers regulating cilia motility in the human respiratory epithelia and the mechanism by which dex and fen modulates CBF.

## Conflict of Interest Statement

The authors declare that the research was conducted in the absence of any commercial or financial relationships that could be construed as a potential conflict of interest.

## Supplementary Material

The Supplementary Material for this article can be found online at http://www.frontiersin.org/Journal/10.3389/fped.2015.00007/abstract
