# Supplementary material for: Dexmedetomidine and Fentanyl Exhibit Temperature Dependent Effects on Human Respiratory Cilia
Source: Front Pediatr. 2015 Feb 11;3:7. doi: 10.3389/fped.2015.00007 (PMC4324059; doi:10.3389/fped.2015.00007)

Effects of dexmedetomidine on ciliary beat frequency in human respiratory epithelia

Nils Welchering[1], Sebastian Ochoa[1], Xin Tian[2], Richard Francis[3], Maliha Zahid[3], Ricardo Muñoz[4], Cecilia W. Lo[3*]

[1] Department of Pediatrics, University of Pittsburgh, Pittsburgh, USA
[2] Office of Biostatistics Research, NHLBI, Washington D.C., USA
[3] Department of Developmental Biology, University of Pittsburgh, Pittsburgh, USA
[4] Department of Critical Care Medicine, University of Pittsburgh, Pittsburgh, USA

*** Correspondence:**
Cecilia W. Lo, PhD
Department of Developmental Biology
University of Pittsburgh
530 45th St. 8120 Rangos Research Center Pittsburgh,
PA 15201, USA
cel36@pitt.edu

1. Supplementary Figures and Tables.

1.1. Suplementary Tables

Supplementary Table 1. Number of obtained videos

		15°C		37°C	
		Number of videos at 5 min	Number of videos at 30 min	Number of videos at 5 min	Number of videos at 30 min
Controls		25	30	34	27
Dex	1nM	44	35	49	40
	5nM	47	38	54	51
	10nM	50	29	48	45
	1nM	44	42	68	61

Fentanyl	5nM	42	44	84	75
	10nM	42	38	85	69
Dex+Fentanyl	1nM	44	52	56	60
	5nM	44	41	68	63
	10nM	39	48	74	62

Supplementary Table 2. Comparison of Mean CBF in all groups at 15°C vs. 37°C at 5 and 30 minutes

		15°C		37°C	
		Mean CBF in Hz at 5 min	Mean CBF in Hz at 30 min	Mean CBF in Hz at 5 min	Mean CBF in Hz at 30 min
Controls		3.53±1.06	3.73±1.46	10.82±1.71	10.42±2.26
Dex	1nM	3.41±1.04	2.57±0.72	11.77±2.54	11.69±2.50
	5nM	2.75±0.79	2.84±0.69	9.83±2.48	10.45±2.45
	10nM	3.12±0.88	3.01±0.84	10.97±3.43	12.17±2.74
Fentanyl	1nM	3.05±0.93	3.07±0.99	10.93±2.46	10.93±2.09
	5nM	2.49±0.65	2.80±0.63	11.10±2.43	11.57±2.32
	10nM	2.72±0.63	3.15±0.97	10.63±1.87	10.44±1.81
Dex+Fentanyl	1nM	2.76±0.80	2.93±0.71	10.99±2.20	11.43±2.00
	5nM	2.82±0.70	2.70±0.72	11.76±2.24	12.34±2.23
	10nM	2.73±0.58	2.85±0.68	11.24±2.03	11.64±1.94

1.2. Suplementary Figures

Supplemental Figure 1: Expression level of adrenergic beta-2 receptor, adrenergic beta-1 receptors, alpha-adrenergic and opioid receptors

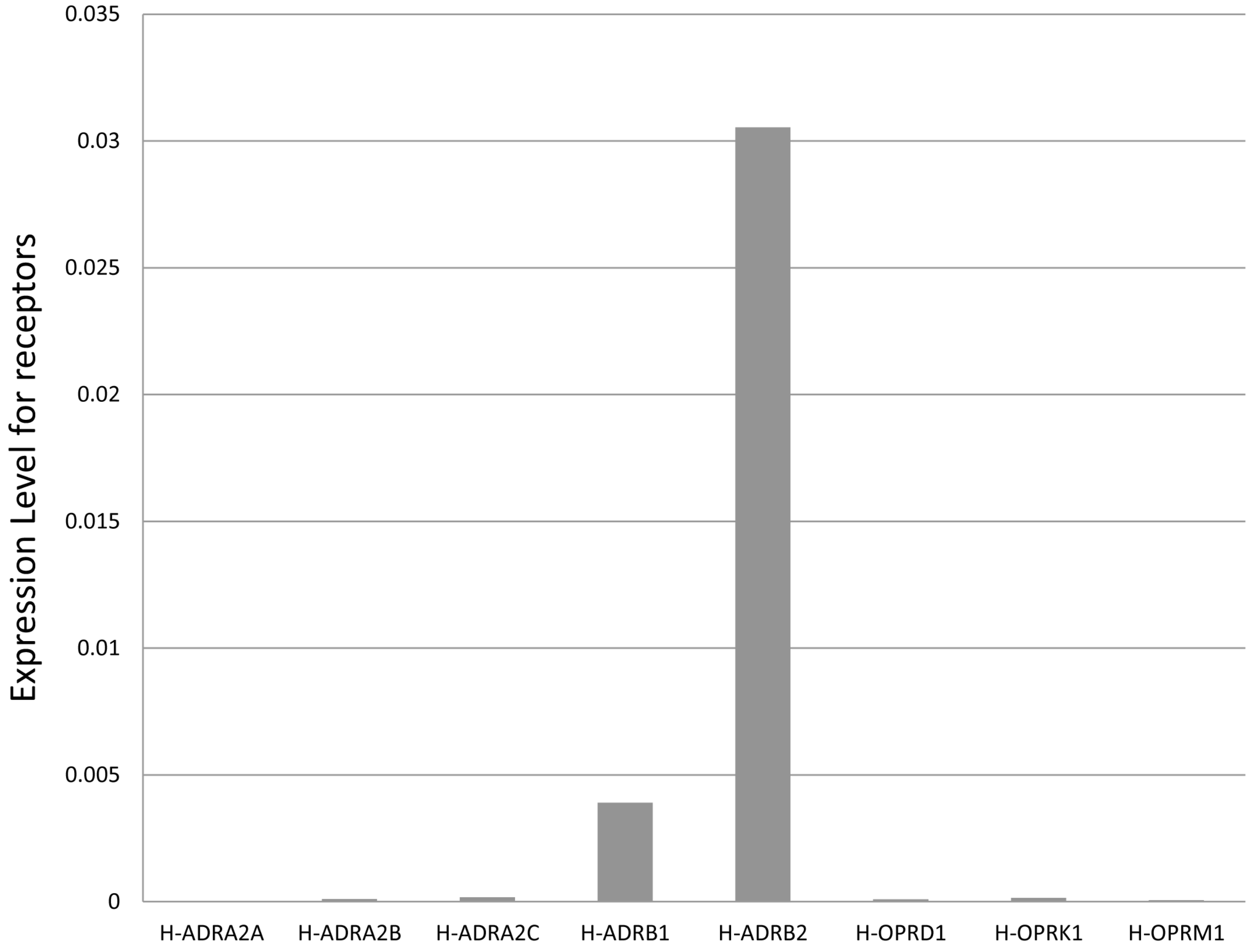

Supplement: Supplementary file 1 [file Data_Sheet_1.PDF]
